# Transmission of Mitochondrial DNA Diseases and Ways to Prevent Them

**DOI:** 10.1371/journal.pgen.1001066

**Published:** 2010-08-12

**Authors:** Joanna Poulton, Marcos R. Chiaratti, Flávio V. Meirelles, Stephen Kennedy, Dagan Wells, Ian J. Holt

**Affiliations:** 1 Nuffield Department of Obstetrics and Gynaecology, University of Oxford, Oxford, United Kingdom; 2 Departamento de Ciências Básicas, Faculdade de Zootecnia e Engenharia de Alimentos, Universidade de São Paulo, Pirassununga, São Paulo, Brazil; 3 MRC Mitochondrial Biology Unit, Wellcome Trust/MRC, Cambridge, United Kingdom; Biomedicum-Helsinki, University of Helsinki, Finland

## Abstract

Recent reports of strong selection of mitochondrial DNA (mtDNA) during transmission in animal models of mtDNA disease, and of nuclear transfer in both animal models and humans, have important scientific implications. These are directly applicable to the genetic management of mtDNA disease. The risk that a mitochondrial disorder will be transmitted is difficult to estimate due to heteroplasmy—the existence of normal and mutant mtDNA in the same individual, tissue, or cell. In addition, the mtDNA bottleneck during oogenesis frequently results in dramatic and unpredictable inter-generational fluctuations in the proportions of mutant and wild-type mtDNA. Pre-implantation genetic diagnosis (PGD) for mtDNA disease enables embryos produced by in vitro fertilization (IVF) to be screened for mtDNA mutations. Embryos determined to be at low risk (i.e., those having low mutant mtDNA load) can be preferentially transferred to the uterus with the aim of initiating unaffected pregnancies. New evidence that some types of deleterious mtDNA mutations are eliminated within a few generations suggests that women undergoing PGD have a reasonable chance of generating embryos with a lower mutant load than their own. While nuclear transfer may become an alternative approach in future, there might be more difficulties, ethical as well as technical. This Review outlines the implications of recent advances for genetic management of these potentially devastating disorders.

## Introduction

One in 400 people carries pathogenic mitochondrial DNA (mtDNA) mutations [1]. These may cause epilepsy, liver failure, cardiomyopathy, or sudden death; or, more commonly, milder disorders such as age-related deafness [1] and/or diabetes [2] and loss of vision [3]. Yet, management and prevention of mtDNA diseases has lagged far behind the genetics revolution [4]. Although preimplantation genetic diagnosis (PGD) has been successfully used to prevent transmission of mtDNA disease [5], [6], its use has been limited for several reasons that are developed in the following sections. Technical improvements in methods for nuclear transfer [7], [8] have aroused expectations of preventing transmission of these disorders, but is this method safe?

## Dose of Mutant mtDNA Determines Severity: Implications for Prenatal Genetic Diagnosis

Chorionic villus sampling (CVS, where early placental tissue is sampled with minimal impact on the foetus) has been extremely successful in preventing recurrence of Mendelian genetic diseases, but not for maternally inherited diseases, caused by mutations in the mtDNA, because of the problem of heteroplasmy [9], [10]. Thousands of mtDNA copies are present in every nucleated cell. Normal individuals are homoplasmic (i.e., virtually all their mtDNA copies are identical), but individuals affected by mtDNA diseases are usually heteroplasmic: most of their tissues and cells have a mixture of both normal and mutant mtDNAs. There is also a threshold effect (tissues function normally unless the proportion of mutant mtDNA rises above a particular level) in most diseases. The level of this threshold varies with both tissue and mutation type, usually in the range 50 to 100% mutant mtDNA, but occasionally as low as 10% [11]. Hence, for many mtDNA mutants, disease might be prevented by selecting embryos or actively lowering the level of mutant mtDNA (for instance by using nuclear transfer). But this is not universally applicable, because some mtDNA diseases are commonly homoplasmic and lack a clear threshold [12].

## Unique Inheritance of mtDNA: Heteroplasmy and the Mitochondrial Bottleneck

Heteroplasmy is one reason why the clinical severity of mtDNA disorders is highly variable and can progress with time. In mtDNA disease patients the level of mutant mtDNA commonly [13], [14] (but not always [15]) falls in blood throughout life (perhaps as a result of selection against detrimental mutant mtDNA within a rapidly dividing population of cells [13], [16]). There are a few case reports suggesting that some types of mtDNA mutant accumulate in non-dividing cells such as muscle [14], [17], [18], where mtDNA turnover is slow [19], and less subject to inter-cellular competition [20]. However, this model explains by no means all of such observations [21]. The progressive change in distribution of some human mutants parallels the dynamic of apparently neutral variants in blood and spleen in an animal model [22] and underlines our inability to define the parameters determining the characteristics that we have loosely termed “detrimental.” The scanty available evidence suggests that there is less segregation in somatic tissues between early embryo and birth than post-natally [10], [23]. However, a major component of the germline segregation during transmission of both human [24] and mouse polymorphisms probably occurs during oogenesis [23], [25], and hence during development of the mother, apparently while she was in utero herself.

Factors that affect segregation of mtDNA variants include the biological fitness of dividing cells, the mutant load, and any differences between wild-type and mutant mtDNA in the rate of replication and degradation. While accumulation of mutant mtDNA can sometimes be attributed to genetic drift [26], consistent segregation towards loss or gain of mutant mtDNA has been widely documented in human cultured cells [27]–[29]. Some mutant mtDNAs exhibit segregation in the opposite direction to that predicted on the basis of selection according to mitochondrial function [28], [30]–[33]. Moreover, biased mtDNA segregation has been demonstrated in solid tissues of mice [22]. Two mouse mtDNA variants were selected in different tissues as a result of differences in genetic background [32], [33], even though neither was associated with a marked functional defect [22], nor a detectable difference in mtDNA replication rate [22]. Because differences in production of reactive oxygen species (ROS) affect mtDNA copy number [34], they may contribute to segregation of heteroplasmic mutants.

Analysis of segregation of mtDNA mutants in tissue culture often uses “cybrid” technology, where mtDNA-free immortalized cells are fused with cytoplasm containing the mitochondria under investigation. Because such cells are aneuploid, some investigators dismiss this model as non-physiological [35]. However, it does indicate that several factors might underpin mtDNA segregation in cell lines, including cellular fitness, replication pausing, ROS production, and mitophagy (preferential breakdown and recycling of regions of the mitochondrial reticulum of organelles containing mutant mtDNAs) [29], [36]–[39]. It is now increasingly possible to test the validity of such hypotheses in whole animals [32], [33].

Genetic counseling of women who are carriers of mtDNA diseases is complex because the dose of mutant mtDNA transmitted to offspring may be determined by the so-called “mitochondrial bottleneck” [40], [41], whereby a small number of mtDNAs become the founders for the offspring. If the number of segregating units (groups of clonal mtDNAs that co-segregate) that become the mtDNA founders of the embryo is small, then large fluctuations may occur in a single generation. Hauswirth and Laipis [42]–[44] suggested that two components to this may occur at different developmental stages. Firstly, there is a massive expansion from ∼100 mtDNA genomes in the earliest stages of oocyte development or primordial germ cell (PGC) to 100,000 or so in the mature oocyte [42]. Mitochondrial DNA barely replicates during the early stages of development [45] and pre-existing mtDNA molecules segregate among the cells of the blastocyst [43], [46]–[49]. This represents a second mechanism contributing to switching in the proportion of mutant mtDNAs, since mtDNAs are progressively partitioned at each cell division, ultimately producing the very few cells that will give rise to the entire embryo (the inner cell mass) [42], [44]. Hence, both clonal proliferation of mtDNA in the developing oocyte and mtDNA segregation during early development contribute to the bottleneck.

## Is the Bottleneck Determined by mtDNA Content in Germ Cell Development? Mouse Studies

**Figure 1:**
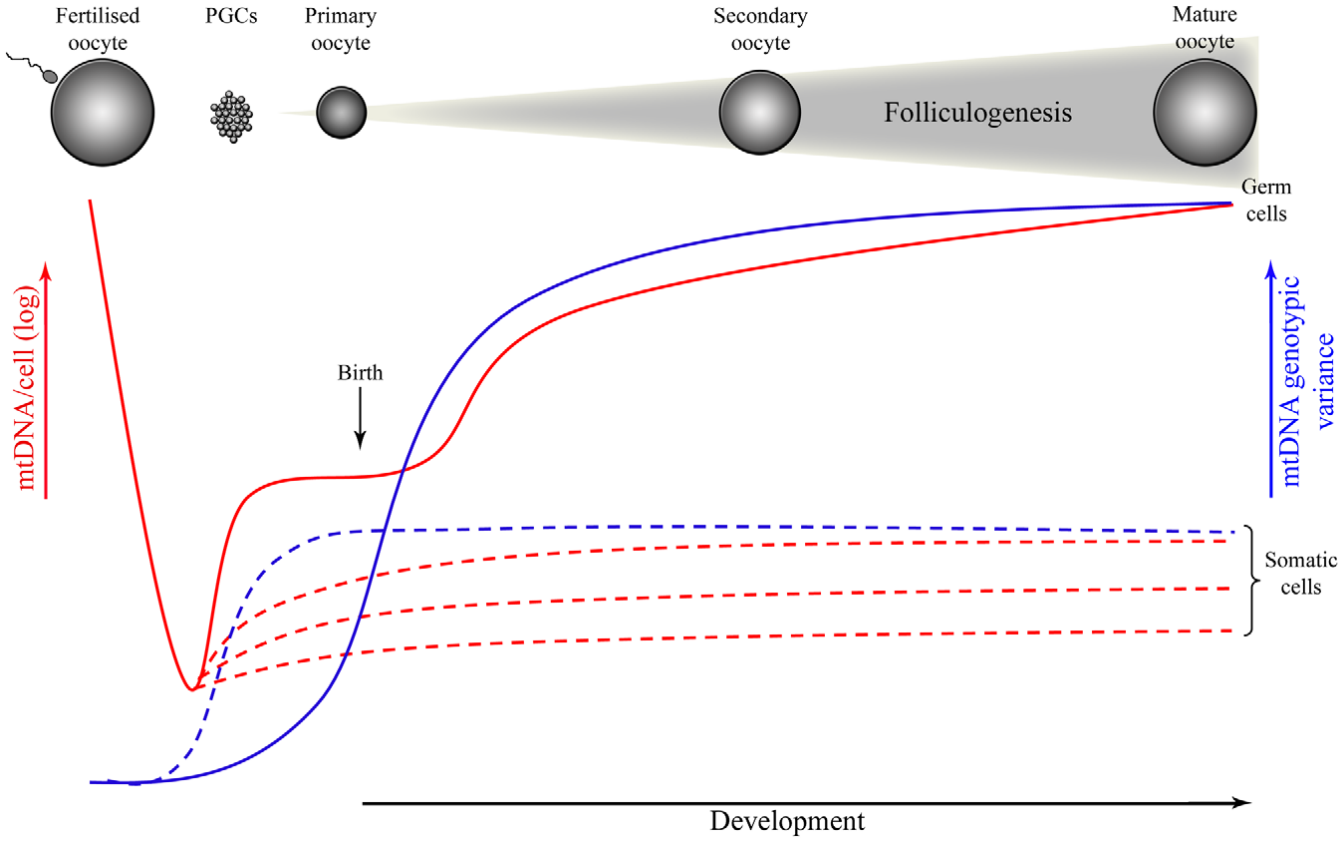
Mitochondrial DNA (mtDNA) copy number and genotypic variance throughout development in germ and somatic cells of mammals. Although mtDNA genotypic variance in somatic cells increases early during development due to cellular differentiation, according to recent findings this will only occur later in germ-line development, during folliculogenesis that takes place after birth. If this is correct, then the mitochondrial genotype of the next generation would be defined only during adulthood, during the folliculogenesis that occurs every cycle of 28 days in women.

Recent studies have carefully quantified mtDNA copy number of individual cells during mouse development [49], [50]–[52]. As predicted [49], [53], the number of mtDNA copies drops to ∼200 molecules in developing PGCs until embryonic day (E) 7.5-8.5 [52], corresponding to the number of segregating units inferred from postnatal analysis [47], [53]. There is, however, conflicting data suggesting that copy number does not fall to values lower than 1,000 in PGCs until E7.5 in mice [50], [51]. As well as depending on technically demanding measurements of the number of mtDNAs in single cells [50], [51], these models have assumed both that segregation in the germline is neutral [54] and that all mtDNA genomes have equal probability of replicating during a single round of cell division. Such assumptions may not be valid, since Wai et al. [52] showed that a sub-population of mtDNAs replicates during folliculogenesis in mice, replenishing the mtDNA content in oocytes and potentially explaining the shifts in mutant load between two generations (Figure 1). While this might explain the variance in mutant load that these authors found in oocytes [52], a more sophisticated analysis demonstrates that a larger set of biological data is needed to establish their claim [55], [56].

In humans, although the meiotic division is initiated in the germline of the developing foetus during the last trimester of pregnancy, primary oocytes remain arrested in the first stage of the meiosis during the years between birth and puberty. In women of reproductive age, a group of oocytes is selected to grow and resume meiosis every cycle of ∼28 days. In most cases this results in the production of a single developmentally competent oocyte. It is possible that clonal expansion of a subpopulation of mtDNA during folliculogenesis in mice (between the stages of primary and mature oocyte) may correspond to the mitochondrial bottleneck [52]. If this is correct, then the segregating unit that is the physical basis of the bottleneck might be the mitochondrial nucleoid, usually containing several mtDNAs [57], rather than a single mtDNA molecule [49]. Understanding the nature of mtDNA packaging in nucleoids would then take on a new importance for biology. On the other hand, if the mitochondrial bottleneck occurs late in germline development, what is the purpose of the dramatic reduction in mtDNA copy number reported during early development? Recent studies have suggested it serves to preserve a homoplasmic population of predominantly healthy mtDNA molecules by selecting against mtDNA mutations that damage mitochondrial function (see below).

## Selection against Detrimental mtDNA Mutants in the Mouse Germline

Three studies suggest that there is selection against detrimental mtDNA mutants in the mouse germline. One group developed a mouse with mtDNA rearrangements modelling Kearns-Sayre syndrome [58] in which the level of mutant mtDNAs in a mother's oocytes fell with time [59]. Like the occasional [60] mtDNA rearrangements that are maternally transmitted [61], these mice had mtDNA duplications in addition to deletions [59].

Another group investigated the transmission of randomly generated mtDNA mutations in a mouse model of mtDNA disease [62]. In this model, there is a mutation in the proof-reading domain of the mtDNA polymerase, *PolgA*, and this generates high levels of point mutations in the mtDNA. The homozygous founder female mice were crossed with wild type and transmitted multiple mtDNA mutations (on average 30 mutations per first generation mouse) to their offspring who were heterozygous for the *PolgA* mutation. Subsequent backcrossing eliminated the mutant *PolgA* allele and hence mtDNA mutants were passively transmitted without generating further mutations. It was thus possible to observe and compare the segregation of multiple different mtDNA mutations in a single lineage. Neutral mutations that do not alter the protein sequence undergo less selection than those that do. Purifying selection can therefore be compared with neutral drift by the relative frequency of such mutations. Clonal selection against deleterious mutations occurred in a remarkably short time frame. Indeed, many deleterious mutations were eliminated within four generations. However, selection was stronger and occurred more rapidly against mutations in genes encoding mRNAs than tRNAs. This may be linked to the apparently high frequency of pathogenic human mtDNA mutations that are identified in tRNAs [62].

A third study, focussing on two pathogenic mtDNA point mutations, again demonstrates selection in mouse [63]. These authors introduced mutant mtDNA from a well-characterised cell line into the germline using cybrid technology and a female embryonic stem cell line. Both the more severe frameshift (insertion) mutation and the milder missense mutation were initially homoplasmic, conferring a severe respiratory chain defect. However, one of the embryonic stem (ES) cell clones became heteroplasmic because a revertant of the frameshift mutation arose; a secondary deletion of the adjacent base restored the reading frame. When this line went into the germline, the mice developed a sub-clinical myopathy and cardiomyopathy but bred normally. The frameshift mutation was lost in favour of the revertant within four generations. None of the offspring had a higher level of the frameshift mutation than their mother, and studies of oocytes showed that the selection had occurred by the time oocytes were mature. These studies are consistent with other studies on mice [52] and on humans [25], [64]. The selection appears to depend on some aspect of mitochondrial function, but studies of the bottleneck have not clarified the precise mechanism or at what stage of oogenesis it is likely to have occurred. While some classic studies in humans [64], [65] and in mice [53] demonstrate that level of mutant mtDNA follows a distribution that may be random [66], others are very different [23], [30]. The latter are skewed towards virtual homoplasmy for both mutant and wild-type mtDNA in oocytes from individual women. One explanation would be that a single mtDNA passes the bottleneck, but there is no obvious mechanism for such an extreme situation. Alternatively this could arise because genetic drift can lead to fixation of neutral mutations [54]. While some investigators consider that the different distributions may be due to the specific mutation, we note that the skewed distributions have only been seen following super-ovulation. Furthermore, close examination of data suggest that the mean level of mutant mtDNA in the oocytes/offspring is not identical with that of the mother, so germline selection [59], [62], [63] is not excluded [54].

But what is the basis of the selection seen in mice and potentially in humans? Only 30% of oogonia established during fetal life develop into matured oocytes, the remainder undergoes apoptosis [67], [68]. Fan et al. [63] suggested that dysfunctional mitochondria generate high ROS levels that are the signal underlying selection against oocytes with high mtDNA mutant load by apoptosis.

A second possible mechanism for selecting against mutant mtDNA is selection at the organelle level. The number of mtDNA copies per mitochondrion in germ cells is thought to be as few as one or two molecules, in comparison to eight or so in somatic cells [57]. Thus, mutations in a few mtDNA copies can be distinguished among wild-type mtDNAs present in the same cell by the effect of mutations on mitochondrial phenotype. For instance, damaged mitochondria might be degraded by intracellular mechanisms such as autophagy or, more specifically, mitophagy [36]. Evidences of this were given by Twig et al. [69] who showed that dysfunctional mitochondria are less likely to fuse with the remaining mitochondria and are degraded by autophagy. Although this event was shown in somatic cells, autophagy is also present in germ cells and early embryos [70] and might be involved in removal of mutant mtDNA from the next generation. Another possibility for selection at the organelle level is competition between dysfunctional and normal mitochondria, where dysfunctional mitochondria might be less efficient for import and enzymatic function of the nucleus-encoded proteins that are required for mtDNA replication. This might result in an advantage of wild-type molecules to replicate over the mutant ones, thus decreasing the mutant load in germ cells and in the next generation [71]. As discussed above, Wai et al. [52] have reported that a sub-population of mtDNAs is replicated during folliculogenesis to replenish the mtDNA content in oocytes. If such a sub-population were positively selected on phenotype by an unknown mechanism, this might explain the observed pattern of selection against mtDNA mutations.

A third possible mechanism is specific to oocytes, based on a structure known as the Balbiani body or the mitochondrial cloud [72]–[74]. The Balbiani body comprises mitochondria and endoplasmic reticulum organized around Golgi elements [73]–[78] that may enable germplasm mRNAs to be specifically inherited by the PGCs in the future embryo. In the same way, a specific mitochondrial sub-population may segregate to the Balbiani bodies and ultimately populate the PGCs [73], [79]–[81], potentially explaining the pattern of selection against severe mtDNA mutations. In some non-mammalian species mitochondria with the highest membrane potentials are found in Balbiani bodies [78], [80], [81], suggesting that high-quality mitochondria and mtDNAs are selected for transmission to the PGCs of the next generation. While this is an appealing mechanism for selecting against mutant mtDNAs, there is little supporting evidence and it is still controversial, even in mouse [52]. Furthermore, the Balbiani body could not explain the progressive decrease in load of mutant mtDNA in mouse oocytes of an individual female with age.

**Figure 2:**
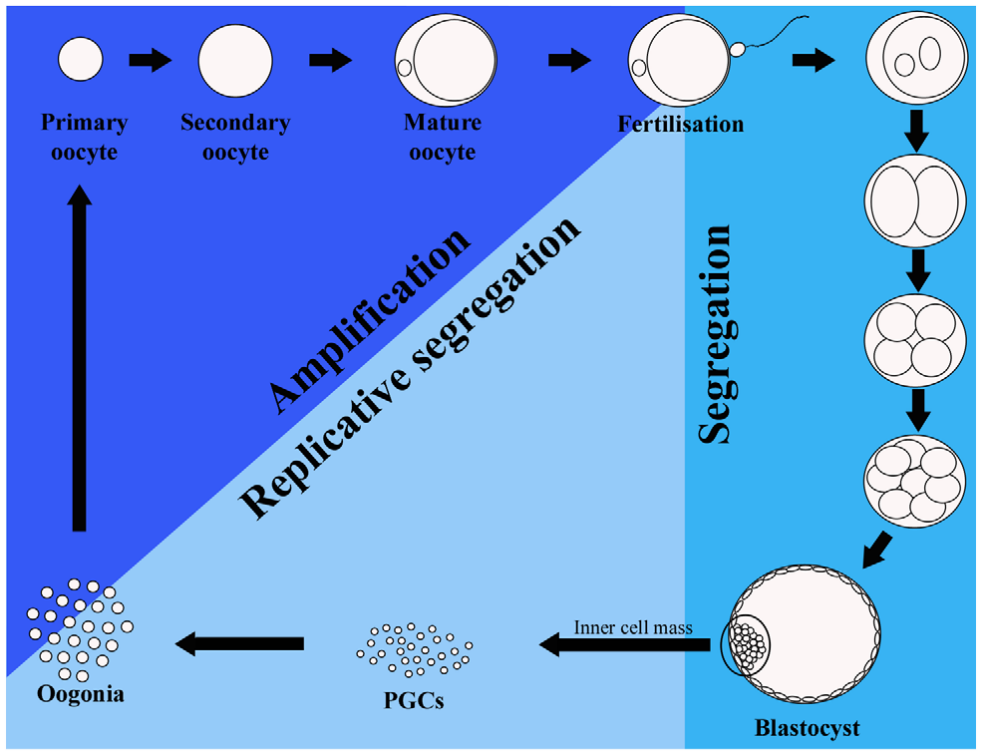
Mitochondrial DNA (mtDNA) cycle in the mouse germline. During early embryo development (“Segregation” on the diagram, representing the first seven to eight days after fertilization) the mtDNA is segregated among daughter cells without being replicated. The number of mtDNA copies thus decreases drastically, being lowest in primordial germ cells (PGCs). The next stage is marked “Replicative segregation,” which implies random replication and partitioning of mtDNAs into daughter cells. The last stage, “Amplification,” is characterized by an exponential amplification of mtDNA molecules. It has been suggested that replication of mtDNA during this stage is restricted to a sub-group of molecules leading to drastic changes in the mtDNA genotype in the mature oocyte. Yet, there seems to be during this stage a selection against mutations in the mtDNA that might occur.

Whatever the underlying mechanism, something occurring during early oogenesis and/or folliculogenesis seems to provide a degree of selection against mutant mtDNA molecules. Studies by Sato et al. [59] and Fan et al. [63] suggest selection occurs during adult life and, therefore, during folliculogenesis, since mutant load drops in mouse oocytes as a function of time (i.e., between two litters). On the other hand, mutations that escape this filter would then be exposed to selection at the level of the individual. Thus, several mechanisms may contribute to the bottleneck and prevent dissemination of mtDNA mutations (Figure 2).

## Mitochondrial DNA Bottlenecks in Human Germ Cell Development

Genetic management of patients with mtDNA disease depends on understanding both germline segregation and the physiological basis of the bottleneck. However, the published human data where oocytes are compared with load in maternal post mitotic tissues are minimal [54]. It is increasingly clear that a major component of this bottleneck has occurred by the time oocytes are mature in human controls [25], patients with mtDNA disease [64], [82], and mouse models [52], [53], [63]. Statistical analysis of oocytes shows that in some cases the distribution of mutant mtDNA is consistent with random drift, but does not exclude the possibility of selection in the germline at an earlier stage [66]. On the other hand, a de novo mutation in a child and in oocytes appeared to be absent from the mother's other tissues [83], suggesting that it arose within the development of her germ cells. Comparison of human and mouse data suggests potentially important differences in both the type of rearrangement that is typical [59], [60] and in the bottleneck size [55]. Hence, it may not be appropriate to extrapolate from the consistent selection against detrimental mtDNA mutants seen in the mouse [62] to humans.

## Implications of Heteroplasmy for Genetic Management of Human Diseases

Oocyte donation would avoid all the problems associated with the presence of mutant mtDNA, but there is a shortage of oocyte donors. Pre-implantation genetic diagnosis for mitochondrial disease could be the best option for patients carrying high levels of mutant mtDNA [6]. This approach involves analyzing embryos produced by in vitro fertilization (IVF) and only transferring those determined to be at very low risk. Preimplantation genetic diagnosis is performed earlier in development (three days after fertilization) than CVS, and two cells are usually taken for mtDNA disease [6]. This is because analysis of one or two cells from an embryo containing 6–10 cells may be more representative of the whole conceptus [84], but not necessarily of the part that will become the foetus. Moreover, sampling two cells rather than only one provides a more confident result (the result from one cell can be compared against the other) and does not appear to impair pregnancy outcome [6], [85].

While PGD clearly has enormous promise for women with sub-clinical levels of mtDNA mutations [6], [23], it may be more complex for women carrying high mtDNA mutation loads and displaying disease symptoms [6], [86]. If such women typically transmit levels of mutant mtDNA close to their own [82], [87], they are likely to produce few if any disease free embryos. If, however, the level of mutant mtDNA in their oocytes were polarized to the two extremes as seen in neuropathy, ataxia and retinitis pigmentosa (NARP) [23], [30], they might have a reasonable chance of usable embryos. This depends to what extent the selection against detrimental mtDNA mutants that is seen in mouse germline also occurs in humans. Nevertheless, offering PGD for certain mtDNA diseases, followed by CVS to confirm that the level of mutant mtDNA in the foetus is low, would likely have advantages over CVS alone. The main drawback of CVS for mtDNA disorders is that it is not entirely certain that the level of mutant mtDNA detectable in a single CVS sample will accurately reflect that of the foetus [10]. Indeed, such data that exist suggest that there is a degree of variation of perhaps ±10% in the level of neutral [88] and pathogenic variants in placenta [83]. Moreover, certain centers are now offering PGD [5], [6], [23].

## Is Nuclear Transfer the Way Forward?

Since Dolly the sheep was created by fusing an adult somatic cell with a recipient enucleated oocyte, producing in Dolly mtDNA inherited not from the somatic cell donor but the recipient oocyte [89], researchers have contemplated altering the mitochondrial population of a human embryo using nuclear transfer. It has been possible to use nuclear transplantation at the zygote stage (pro-nuclear transfer) to partially correct respiration defects and mitochondrial diseases in mice carrying a large-scale deletion of mtDNA [90].

Recently, Tachibana et al. [8] transferred nuclei at an earlier stage; spindle-chromosomal complexes were removed from mature monkey oocytes, with minimal if any adherent mtDNA, and placed into other oocytes from which the complex had been removed. This study resulted in the generation of three healthy offspring with less than 3% of nuclear donor mtDNA [8]. More recently, Craven et al. [7] transferred pro-nuclei between human zygotes resulting in minimal carry-over of nuclear donor mtDNA and compatible onward development to the blastocyst stage in vitro. Because of the current regulations and the paucity of “spare” human embryos, this study was carried out in abnormally fertilized embryos. Disappointingly, the levels of nuclear donor mtDNA were very variable between cells of the resulting embryos (ranging from less than 0.5 to 11.4%), suggesting that mtDNA segregation might be disturbed by the procedure. This may be a consequence of using genetically abnormal embryos that would not occur in bona fide treatment cycles. But it might be because they used a drug that specifically targets the microtubule-based system (nocodazole) for organizing mitochondria in the cell. Despite this, both studies [7], [8] (with their pros and cons) are of fundamental importance and hold promise for the future treatment of mtDNA diseases.

A different procedure, ooplasm donation (cytoplasm from a donor oocyte), offers an alternative [91]. Ooplasm donation has been used in humans as a treatment for poor IVF embryo development for a type of infertility that might be due to intrinsic defects of the oocyte cytoplasm. In this experimental procedure, mitochondria, cytoplasm, and associated structures from a donor oocyte are injected into a recipient unfertilised oocyte prior to IVF. Mitochondrial DNA analysis of children born following the procedure demonstrated that the contribution of donor mtDNA is small [92], but, in some cases, the proportion of donor mtDNA far exceeded the expected 10–15% [93], based on the volume of cytoplasm derived from the donor. While genetic drift might occasionally underlie such a change, experiments on bovine zygotes suggest that mitochondrial replacement can be consistently improved by centrifugation and removal of the recipient mtDNA without apparent effects on development [94], [95]. Centrifugation causes mitochondria to concentrate in one of the zygote's poles [94], [95], allowing removal of mitochondrion-enriched cytoplasm by micromanipulation. Doing this, it is possible to remove over 60% of recipient-zygote mtDNA before ooplasmic transfer [94]. Furthermore, the use of purified mitochondria as donor mtDNA [96]–[98] might decrease the mutant load to low levels, ultimately avoiding transmission of the mitochondrial disorders.

Will any of these procedures be viable alternative strategies to more conventional genetic management? Nuclear transfer sounds simple and seems effective in mice [90], monkeys [8], and in human pre-implantation embryos [7], yet there remain very many unknowns. Mitochondrial DNA encodes only a handful of proteins, the remainder of the thousand or so proteins that go to make up the mitochondrion being encoded by the nucleus. This arrangement necessitates nucleo-mitochondrial interactions, which are as yet poorly understood. In embryos derived either by nuclear transfer or ooplasm donation, the genetic material originates from three unrelated parents (two providing the nucleus and one the mtDNA). While extreme (non-physiological) mismatch between nuclear and mitochondrial DNA has clearly deleterious effects on nucleo-mitochondrial interactions [99], [100], might subtle errors in these interactions occur following nuclear transfer? The consequences of uncoupling the mitochondria and nucleus, followed by the introduction of DNA from an unrelated individual are unknown. Genetic studies of such interactions strongly suggest that major problems are unlikely [32], [33]. However, backcrossing mice so that one mtDNA was substituted for another on a standardized nuclear background can alter either physical [101] or cognitive performance [102] and even the anatomy of the brain [102]. Furthermore, studies on mice suggest that mtDNA carried-over with the nuclear DNA of the donor zygote (karyoplast) may be replicated faster than that of the recipient, perhaps depending on its proximity to the nucleus [103]. Since nuclear transfer experiments in multiple species show that donor mtDNA may persist in embryos and tissues from the offspring [104], one cannot assume that the mitochondria from the “healthy” enucleated oocyte will ultimately outnumber the mutant mitochondria in the tissues of the foetus and child. Furthermore, even in the best hands, the success rate of achieving a pregnancy per egg is low and donor oocytes are scarce.

## Conclusion

In conclusion, the many ethical, scientific, and pragmatic problems have been a major impediment in the genetic management of mtDNA diseases. Recent experiments on animals suggest that nuclear transplant holds future promise. Currently, the most ethical course of action may be to weigh-up the uncertainties and use new approaches such as PGD in an attempt to help these distressed families.
